# A *Bacillus* Phage From Bangladeshi Soil, Phage APU1: Genomic Characterisation and Comparative Genomic Analysis

**DOI:** 10.1111/1758-2229.70415

**Published:** 2026-09-17

**Authors:** Arefeen Haider, Tasnuva Jahan Esha, Sezanur Rahman, Jannat Akther, Naimul Islam Faysal, Tahsin Khan, Shakhinur Islam Mondal, Anowara Begum, Sudhangshu Kumar Biswas, Mustafizur Rahman, Mohammad Jubair

**Affiliations:** ^1^ Genome Centre, Infectious Diseases Division Icddr,b Dhaka Bangladesh; ^2^ Biotechnology Program, Department of Biochemistry & Microbiology, School of Health & Life Sciences North South University Dhaka Bangladesh; ^3^ Virology Laboratory, Infectious Diseases Division Icddr,b Dhaka Bangladesh; ^4^ Department of Genetic Engineering and Biotechnology Shahjalal University of Science Technology Sylhet Bangladesh; ^5^ Department of Microbiology University of Dhaka Dhaka Bangladesh; ^6^ Bacteriophage Biology and Genomics Lab, Department of Biotechnology and Genetic Engineering Islamic University Kushtia Bangladesh

**Keywords:** *bacillus* bacteriophage, comparative genomics, soil virome

## Abstract

*Bacillus* species are environmentally resilient bacteria associated with soil, food, industrial settings and opportunistic infections, yet their bacteriophages remain incompletely characterised, particularly from underexplored environmental sources. We isolated and characterised phage APU1, a *dsDNA* phage from agricultural soil in Bangladesh. Partial 16S rRNA gene sequencing identified the enrichment host conservatively as *Bacillus* sp. APU1 has a 153,847 bp genome that encodes 236 predicted proteins, including 84 shorter than 100 amino acids. CheckV classified the genome as high‐quality, with an estimated completeness of 99.91% and no detectable host‐gene contamination. Functional annotation identified genes associated with DNA replication, structural assembly, packaging and host lysis. PhaBOX classified APU1 as virulent, with PhaTYP and CHERRY prediction scores of 1.0. CARD/VFDB screening did not detect CARD‐listed antimicrobial resistance genes or VFDB‐listed virulence factor genes. Comparative genomic analyses using VICTOR, VIRIDIC and VIPTree placed APU1 within a group of related *Bacillus* phages. APU1 shared 89.7%–89.8% intergenomic similarity with its closest relatives, supporting its classification as a genomically distinct phage within this group. This study expands the genomic catalogue of *Bacillus‐*associated phages from Bangladeshi soil and provides a foundation for future studies on phage diversity, host range, morphology and biological properties.

## Graphical abstract

Phage APU1, isolated from Bangladeshi agricultural soil, is a high‐quality, computationally predicted lytic *Bacillus* phage with a 153,847‐bp genome and no detected antimicrobial resistance or virulence factor genes. Comparative genomics identified APU1 as a genomically distinct member of a related *Bacillus* phage group.

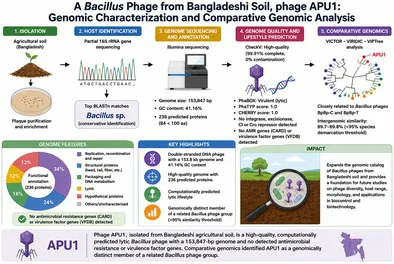

## 1 Introduction

Bacteriophages (phages) are viruses that infect and replicate within bacteria via either a lytic or lysogenic cycle (Yuan et al. 2015). They are abundant in nearly all environments and play essential roles in regulating bacterial populations, shaping microbial community dynamics and driving horizontal gene transfer (Batinovic et al. 2019). Unlike chemical antimicrobials, phages are highly specific and do not harm plants, animals, humans or non‐target microbes (Huang et al. 2024), making them attractive candidates for therapeutic and biotechnological applications. These include antibacterial therapies, vaccine delivery, phage display systems, food safety biocontrol and bacterial typing (Kusmiatun et al. 2015; Lorenz et al. 2013). Thus, phages are considered a core part of microbial ecosystems, shaping bacterial communities and influencing microbial evolution (Hayes et al. 2017).

Soil viromes remain particularly underexplored despite estimates suggesting that up to 97% of viruses reside in solid matrices like soil and sediment, whereas only 1.8% of publicly available viral metagenomes are soil‐derived. Investigating soil viromes can provide insight into the largely untapped phage diversity and their roles in ecological processes (Goller et al. 2020). While phage genomes are typically small and easy to sequence compared to bacterial genomes, they are often underrepresented in microbiome studies (Hayes et al. 2017). This omission may bias conclusions and limit our understanding of virus‐host interactions and viral contributions to microbial community structure.

The genus *Bacillus* comprises diverse species, including *Bacillus licheniformis* , *Bacillus subtilis* , and *Bacillus pumilus* , some of which are linked to foodborne illnesses (Lorenz et al. 2013). *B. pumilus* , in particular, has been implicated in infections affecting plants, animals and humans (Bentur et al. 2007; Shah et al. 2019). This species exhibits high resistance to extreme stressors such as ultraviolet radiation, ionising radiation, high salt concentrations and it readily forms biofilms, complicating its removal from medical and industrial environments (Cortesao et al. 2019; Zhu et al. 2020). Notably, *B. pumilus* has shown resistance to concentrations of hydrogen peroxide up to three times higher than those tolerated by other *Bacillus* species (Lorenz et al. 2013). These attributes make it difficult to control using conventional approaches. Phages and their lytic enzymes have been proposed as promising alternatives for targeting *Bacillus* sp., particularly for eradicating spores and biofilms (Fu et al. 2020; Skorynina et al. 2022).

Previous research on bacteriophages infecting *Bacillus* sp. reveals a diverse collection of viruses with varied morphologies and genomic characteristics, highlighting their role in bacterial ecology and evolution. Early work established foundational knowledge through the isolation and basic characterisation of phages (Badran et al. 2018), while more recent genomic studies have uncovered significant diversity. For instance, the novel temperate phage vB_BpuM‐ZY1, isolated from mangrove sediments, is a myophage with a 37 kb genome and has been proposed to represent a new genus within the Peduoviridae family due to its low genomic similarity to known phages (Jin et al. 2024). In contrast, the large myovirus phiAGATE has a 150 kb genome and prompted a re‐evaluation of the taxonomy within the Spounavirinae subfamily (Barylski et al. 2014).

Despite this importance, *Bacillus‐*associated phages remain poorly characterised. Only around a 100 plus complete genomes of *Bacillus* phages have been deposited in GenBank (Chen et al. 2023). The diversity and host range of *Bacillus* phages remain largely unknown (Fard et al. 2010), and phages of this host are underrepresented in both culture‐based and metagenomic studies. While several phages infecting *Bacillus* sp. have been sequenced, including Bp8p‐C, Bp8p‐T and Bobb (Asare et al. 2015; Yuan et al. 2015), many have not been assessed at the species level using modern genomic and phylogenomic tools. Advances in sequencing technology and library preparation methods have expanded the field of phage genomics (Casey et al. 2021), and whole‐genome sequencing (WGS) using DNA library‐based approaches remains the most effective strategy for characterising phages (Fard et al. 2010). Moreover, metagenomic analyses offer powerful tools for investigating uncultured viral populations and their evolutionary relationships (Hayes et al. 2017). However, databases such as IMG/VR (Camargo et al. 2022) and GOLD (Mukherjee et al. 2019) can facilitate mapping new phages to their environmental contexts. These tools offer metadata on genome completeness, predicted host lineage and ecological origin, providing a framework for exploring viral environmental distribution and host associations across terrestrial biomes.

To address these gaps, we isolated and characterised APU1 phage, a predicted lytic *dsDNA* phage targeting *Bacillus* sp., from an environmental soil sample. Our investigation utilised whole‐genome sequencing to determine its genomic architecture, followed by functional annotation to identify putative genes and metabolic pathways. Comparative genomic analyses were conducted to assess taxonomic relationships with other *Bacillus*‐infecting phages.

## 2 Materials and Methods

### 2.1 Sample Collection and Phage Isolation

Soil samples were collected in 2024 from a dairy farm in Dhaka, Bangladesh, using sterile containers (Thermo Fisher Scientific), transported on ice and processed within 3 h. For phage enrichment, 5 g of soil was mixed with 10 mL of phage buffer (68 mM NaCl, 10 mM MgSO_4_, 10 mM Tris‐Cl, pH 7.5), and 5 mL of log‐phase *Bacillus* sp. culture (Genome Centre biobank, icddr,b) grown in LB containing 10 mM MgSO_4_ and 1 mM CaCl_2_. The mixture was incubated at 37°C for 24 h (180 rpm), centrifuged (10,000 × g, 10 min), and filtered through a 0.22 μm membrane (Millipore). Phage presence was confirmed using the double‐layer agar technique, with LB agar (1.5%) and LB soft agar (0.7%) containing filtrate and host culture (Kropinski et al. 2009). After overnight incubation (37°C), plaques were assessed for clarity, circularity and halo formation. Serial 10‐fold dilutions of high‐titre APU1 lysates were plated and incubated at 37°C for 16–18 h. Lytic activity was further observed by spotting 10 μL of lysate (~10^8^ PFU/mL) onto fresh bacterial lawns under the same conditions. The bacterial enrichment host used for APU1 isolation was identified by partial 16S rRNA gene sequencing. A pure bacterial colony was grown on Mueller‐Hinton agar at 37°C for 24 h, and genomic DNA was extracted using the DNeasy Blood & Tissue Kit according to the manufacturer's instructions. The 16S rRNA gene was amplified using universal primers 27F and 1492R (Weisburg et al. 1991) with the GoTaq Flexi DNA Polymerase Kit in a 25 μL reaction. Thermal cycling consisted of initial denaturation at 95°C for 2 min; 30 cycles of 94°C for 30 s, 57°C for 30 s and 72°C for 1 min; and a final extension at 72°C for 10 min. PCR products were confirmed on a 1.5% agarose gel in TBE buffer, purified using ExoSAP‐IT, and sequenced using the BigDye Terminator v3.1 Cycle Sequencing Kit on an ABI 3500xL Genetic Analyser. Sequence reads were quality‐checked, trimmed, and assembled into a consensus sequence using Chromas. The consensus sequence was compared against the NCBI nucleotide database using BLASTn (Camacho et al. 2009), and host identification was assigned based on percent identity, query coverage, E‐value, and taxonomic consistency among the top BLASTn hits.

### 2.2 DNA Extraction, Sequencing and Genome Assembly

A single plaque was excised and eluted in SM buffer (50 mM Tris–HCl, 100 mM NaCl, 8 mM MgSO_4_, pH 7.5), followed by three rounds of plaque purification to ensure clonal purity. High‐titre phage lysates (~10^9^ PFU/mL) were generated by infecting log‐phase host bacteria at an MOI of 0.01, incubating until complete lysis (6–8 h), and concentrating the particles via PEG8000 precipitation (Yamamoto et al. 1970). DNA was extracted using the DNeasy Blood & Tissue Kit (Qiagen, Germany), incorporating an RNase A treatment (10 μg/mL for 30 min at 37°C) to remove residual RNA. DNA concentration and purity were evaluated by Nanodrop spectrophotometer (A260/A280 > 1.8) and Qubit 4.0 fluorometer. Sequencing libraries were prepared using the Illumina DNA Prep Kit, followed by paired‐end sequencing (2 × 250 bp) on an Illumina MiSeq platform with a 500‐cycle kit. Raw sequencing reads were first quality‐assessed using FastQC v0.11.9 (Andrews 2010) and trimmed using Trimmomatic v0.39 with the parameters SLIDINGWINDOW:4:20, HEADCROP:20, CROP:220 and MINLEN:150 (Bolger et al. 2014). High‐quality reads were assembled de novo using SPAdes v3.15.5 (Bankevich et al. 2012) with the options—careful and—only‐assembler, applying a range of *k*‐mer values (21, 33, 55, 77, 87, 99, 111, 119, 127). Assembly quality was assessed with QUAST v5.2.0 (Gurevich et al. 2013), and read coverage depth was calculated using BBmap v37.62 (Bushnell 2015). The contig with the highest read depth was retained as the APU1 genome assembly for downstream analysis.

### 2.3 Genome Annotation, Host Prediction, and Lifestyle Analysis

Genome annotation was conducted using Pharokka v1.7.3 (Bouras et al. 2023). In parallel, PhageScope (Wang et al. 2023) was used to integrate genome assembly validation, lifestyle prediction, host assignment and annotation in one pipeline. Genome completeness was assessed using CheckV v1.0.1 (Nayfach et al. 2021), following MIUViG standards (Roux et al. 2019). Coding sequences (CDSs), tRNAs (via ARAGORN v1.2.38) (Laslett and Canback 2004) and transmembrane helices (via TMHMM v2.0) (Krogh et al. 2001) were identified. All 236 predicted CDSs from the final PhageScope‐supported annotation were retained, including predicted proteins shorter than 100 amino acids. InterProScan 5 (Jones et al. 2014) was used for conserved‐domain and protein‐match analysis, and detailed interpretation of hypothetical proteins was focused on proteins longer than 100 amino acids or proteins with identifiable conserved‐domain evidence to avoid overinterpretation of low‐confidence annotations. Computational host prediction was carried out using the PhageScope host‐assignment module and was interpreted separately from the experimental partial 16S rRNA gene‐based identification of the enrichment host. Lifestyle classification was inferred through PhaBOX suite (PhaTYP, CHERRY, PhaVIP and PhaMer) (Shang et al. 2023). In addition, the final genome annotation was manually inspected for genes commonly associated with temperate phages, including integrase, excisionase, CI/Cro repressors, recombinases and other lysogeny‐associated proteins. Predicted proteins were screened against CARD and VFDB through the PhageScope workflow using MMseqs‐based homology searches (Hauser et al. 2016; Alcock et al. 2023; Liu et al. 2022). All outputs were cross‐checked for consistency across platforms, and the final dataset—comprising annotated genes, functional domains, tRNA profiles and lifestyle classification—served as the foundation for subsequent comparative genomic analyses.

### 2.4 Comparative Genomic and Phylogenetic Classification

The evolutionary relatedness of phage APU1 was determined through comparative genomic analysis with 19 known *Bacillus*‐infecting phages retrieved from the INPHARED database (non‐redundant, > 30 kbp, > 40 CDSs, Caudoviricetes). Whole‐proteome distances were calculated in VICTOR (GBDP, D6 formula) (Meier‐Kolthoff and Goker 2017) to generate a phylogenetic tree, while VIPTree (Nishimura et al. 2017) provided complementary tBLASTx‐based proteomic clustering. Pairwise intergenomic average nucleotide identity (ANI) values were computed with VIRIDIC (Moraru et al. 2020) to define species‐ and genus‐level relationships.

## 3 Results

### 3.1 Isolation and Plaque Morphology of Phage APU1

**FIGURE 1:**
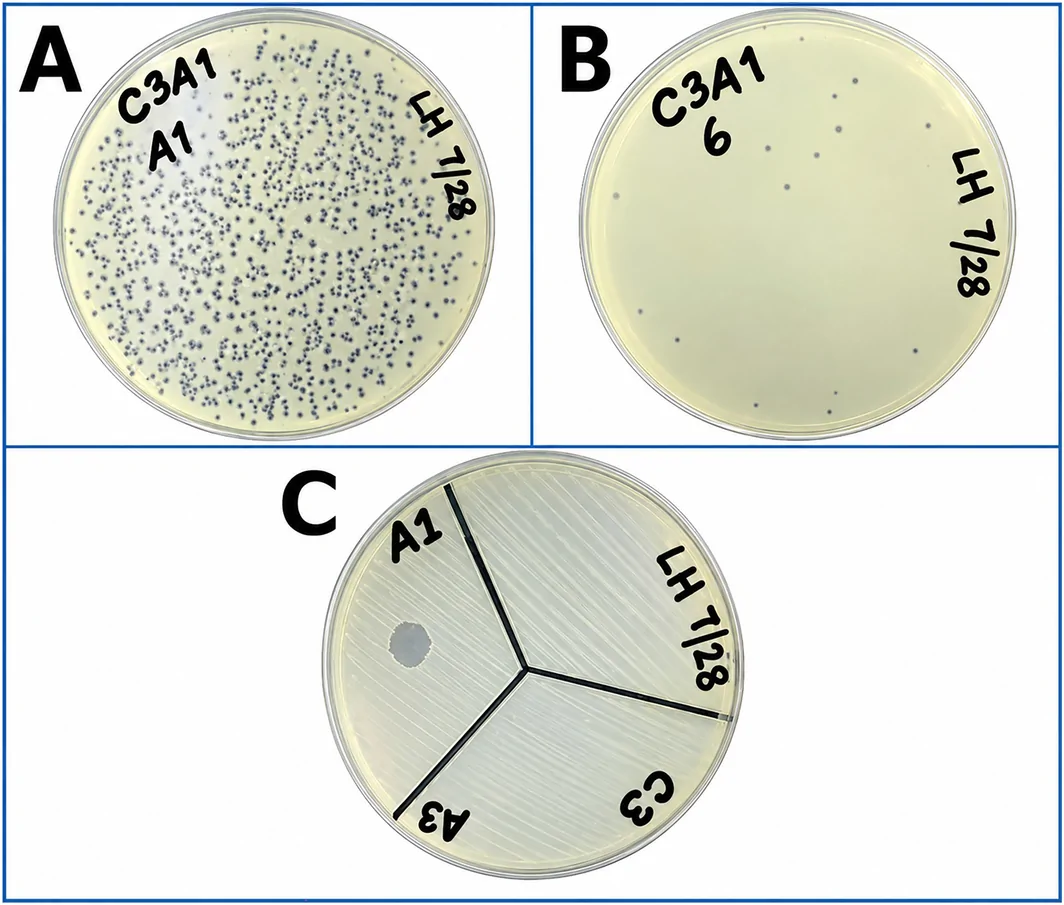
Plaque morphology and lytic activity of phage APU1. (A and B) Plaque formation on *Bacillus* sp. double‐layer agar plates at different dilutions of the enriched lysate. Clear, circular plaques (0.5–1.5 mm in diameter) were observed after 16–18 h incubation at 37°C. (C) Spot test showing a distinct lysis zone, suggesting lytic activity of phage APU1 against the *Bacillus* sp. host under the assay conditions.

Phage APU1 was isolated from soil collected at a dairy farm in Dhaka, Bangladesh, using *Bacillus* sp. as the enrichment host. After incubation and filtration, phage activity was indicated by the formation of distinct plaques on double‐layer agar plates. Plaques were circular, uniformly distributed and clear with well‐defined boundaries, around 0.5–1.5 mm in diameter (occasional larger coalesced plaques at low dilution) after 16–18 h of incubation at 37°C (Figure 1). Morphological characteristics remained consistent through successive purification rounds, indicating clonal stability. Spot tests using high‐titre lysates (~10^8^ PFU/mL) produced complete lysis zones on fresh *Bacillus* lawns, suggesting lytic activity against the tested enrichment host under assay conditions. Among several plaques obtained from the initial enrichment, the phage exhibiting the most consistent and clear lytic activity was selected for further genomic characterisation and designated APU1.

Partial 16S rRNA gene sequencing of the enrichment host generated a 788‐bp consensus sequence. BLASTn analysis placed the isolate within the genus *Bacillus*. The closest matches included *Bacillus xiamenensis*, *Bacillus altitudinis* and *Bacillus aerophilus* , each showing 100% query coverage, an *E*‐value of 0 and 99.62% sequence identity. Because multiple *Bacillus* species produced equivalent top BLASTn matches, the enrichment host was conservatively identified as *Bacillus* sp.

### 3.2 Genome Sequencing, Assembly and General Features of Phage APU1

**FIGURE 2:**
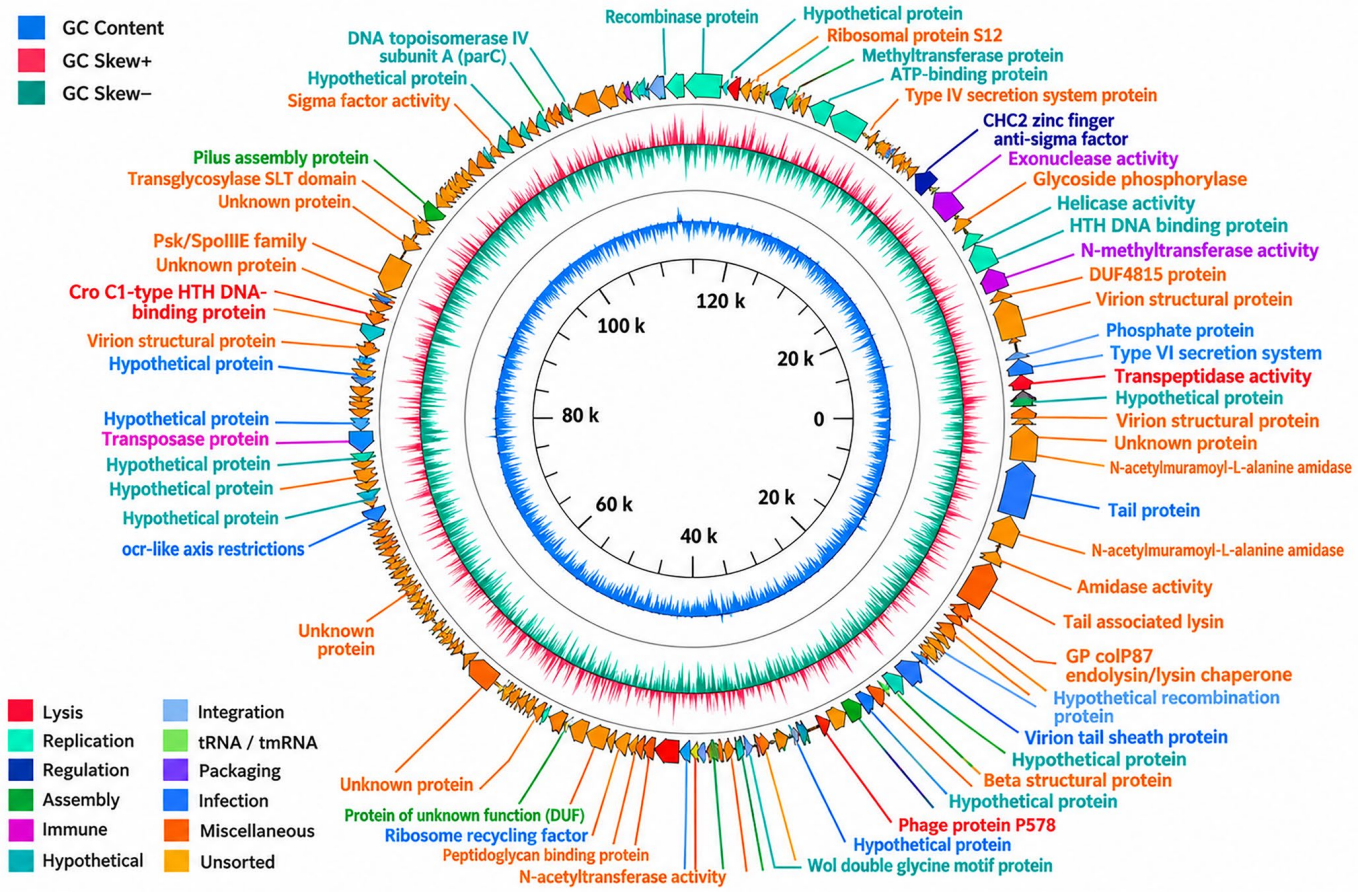
Circular visualisation of the linear *dsDNA* genome of phage APU1. The outer tracks show predicted coding sequences on the forward and reverse strands, with arrows indicating gene orientation. Coding sequences are coloured according to functional category, including structural proteins, DNA replication/packaging‐associated proteins, lysis‐associated proteins and hypothetical proteins. The inner tracks show GC content and GC skew across the 153,847 bp genome.

Phage APU1 possesses a 153,847 bp linear dsDNA genome with 236 predicted coding sequences and a GC content of 41.16%. The genome exhibits a modular architecture with predicted functional clusters for replication, structural components and lysis functions (Figure 2). CheckV classified the APU1 genome as high‐quality according to the MIUViG criteria. The assembled genome was 153,847 bp in length and had an estimated completeness of 99.91% based on the AAI‐based high‐confidence method. CheckV estimated 0.0% contamination and detected no host genes. Annotation using ARAGORN identified five tRNA genes (two tRNA‐Met(cat), one tRNA‐Tyr(gta), one tRNA‐Phe(gaa) and one tRNA‐Asn(gtt)), but no tmRNA. TransTermHP (Kingsford et al. 2007) predictions revealed 36 Rho‐independent transcriptional terminators, suggesting tightly regulated transcriptional units across the genome. The genome encodes key functional proteins, including tail‐associated lysins, peptidoglycan hydrolases and terminase‐like packaging proteins. No transmembrane helices were detected in any predicted proteins by TMHMM analysis. The circular genome visualisation map (Figure 2) visualises these features, including gene directionality, functional categories, GC content and GC skew patterns derived from integrated PhageScope and Pharokka annotations.

### 3.3 Genome Annotation, Functional Prediction and Lifestyle Analysis

Genome annotation revealed 236 predicted coding sequences (CDSs), all of which were retained in the final PhageScope‐supported annotation, including 84 predicted proteins shorter than 100 amino acids. The complete CDS annotation is provided in Table S1. InterProScan identified conserved‐domain/protein‐match hits in 70 predicted proteins associated with key phage functions. These included structural components such as portal proteins (IPR006944) and prohead serine proteases (IPR054613), host‐interaction elements including tail spike hydrolases and peptidoglycan amidases (IPR002502) and regulatory proteins characterised by ATPase and helix‐turn‐helix motifs. Gene Ontology terms supported these findings, with annotations predominantly associated with virion structure and infection‐related processes. For conservative interpretation, detailed discussion of hypothetical proteins was focused on proteins longer than 100 amino acids and those with identifiable conserved‐domain evidence. Among these proteins, phage‐associated domains such as lysozyme‐like hydrolases and baseplate components were detected, suggesting possible uncharacterised structural or enzymatic roles. Detailed InterProScan/domain‐hit results are provided in Table S2.

No recognisable auxiliary metabolic genes were annotated using the applied PhageScope‐supported workflow. In addition, CARD/VFDB screening through the PhageScope workflow using MMseqs‐based homology searches did not detect CARD‐listed antimicrobial resistance genes or VFDB‐listed virulence factor genes under the applied thresholds.

PhaBOX classified APU1 as virulent, with PhaTYP and CHERRY prediction scores of 1.0. Manual inspection of the final genome annotation did not identify canonical integrase, excisionase, CI repressor or Cro repressor genes. Therefore, the lifestyle assignment was interpreted as computational evidence supporting a predicted lytic lifestyle. The computational host prediction was carried out using the PhageScope host‐assignment module and was interpreted separately from the experimental partial 16S rRNA gene‐based identification of the enrichment host (Table 1). The PhaVIP module assigned annotations to 224 of 236 predicted CDSs, corresponding to an annotation rate of 95%, supporting the completeness of the functional annotation.

**TABLE 1:** PhaBOX output showing computational lifestyle classification, host prediction and annotation summary for phage APU1.

Genome ID	Genome length (bp)	Predicted lifestyle	PhaTYP score	Computational host prediction	CHERRY score	Host prediction method	Predicted genus	Genes cluster	Annotation rate	Predicted lineage
*Bacillus* phage APU1	153,847	Virulent	1	*Bacillus* sp. TH007	1	CRISPR‐based (DB)	*Agatevirus*	Known genus	95% (224/236 genes)	Duplodnaviria > Caudoviricetes > *Agatevirus*

### 3.4 Comparative Genomic and Phylogenetic Analyses

**FIGURE 3:**
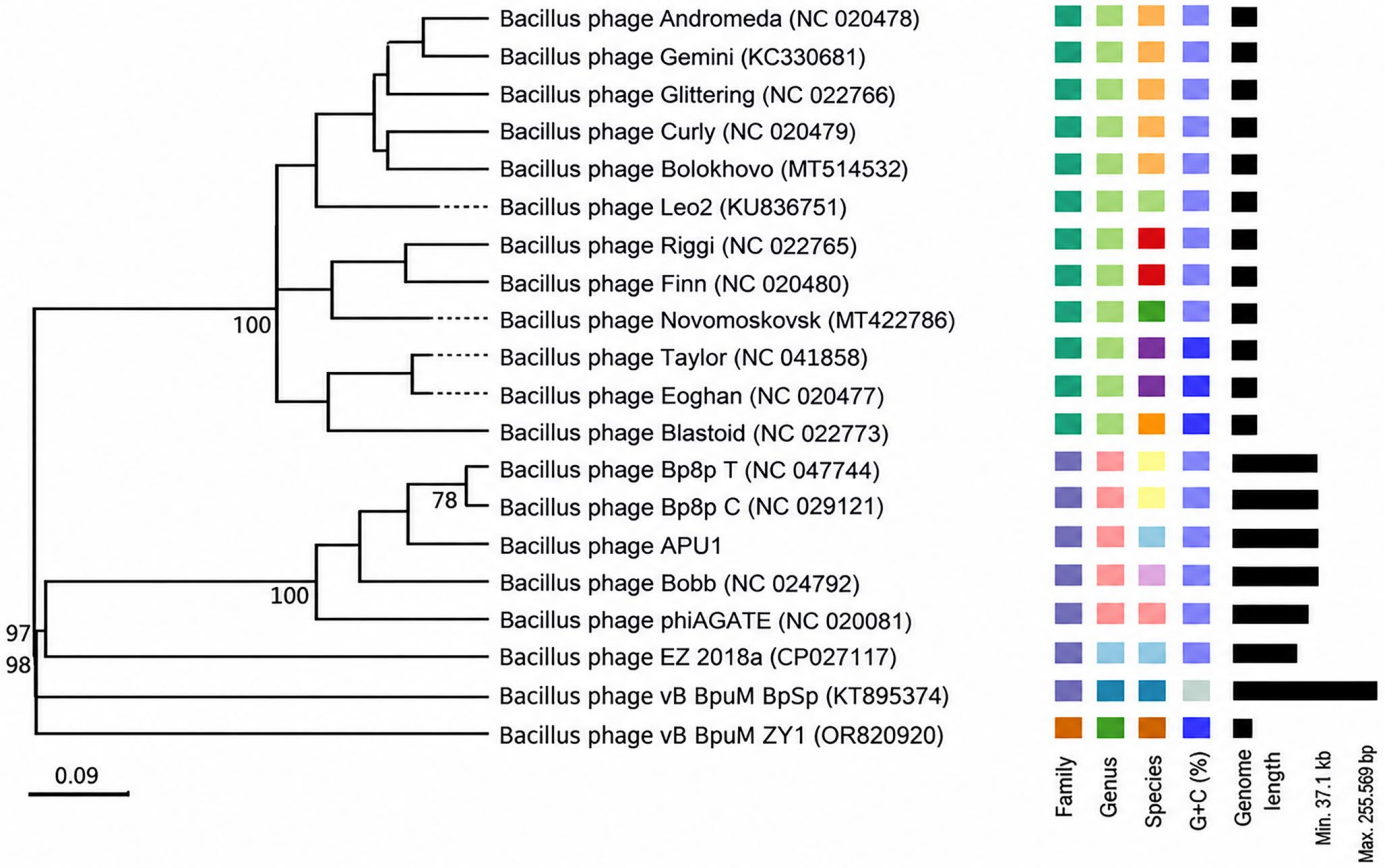
Phylogenomic tree of phage APU1 and related *Bacillus* phages generated by VICTOR (GBDP, D6 metric). APU1 clusters within genus‐level Cluster 5 alongside *Bacillus* phages *phiAGATE*, *Bobb*, *Bp8p‐C* and *Bp8p‐T*. Branch lengths indicate genome‐based distances (scale bar: 0.2 substitutions/site), with bootstrap values > 90% supporting major nodes.

Whole‐proteome distance analysis using VICTOR positioned phage APU1 within genus‐level Cluster 5 (Figure 3), alongside *Bacillus* phages phiAGATE, Bobb, Bp8p‐C and Bp8p‐T. This clade exhibits consistent *Bacillus* species host specificity and comparable genome sizes (150–160 kbp range), with high proteome similarity (D6 < 0.3) supporting their classification within a shared genus. Within this group, APU1 formed an independent branch, indicating genomic differentiation while maintaining overall relatedness to the cluster.

**FIGURE 4:**
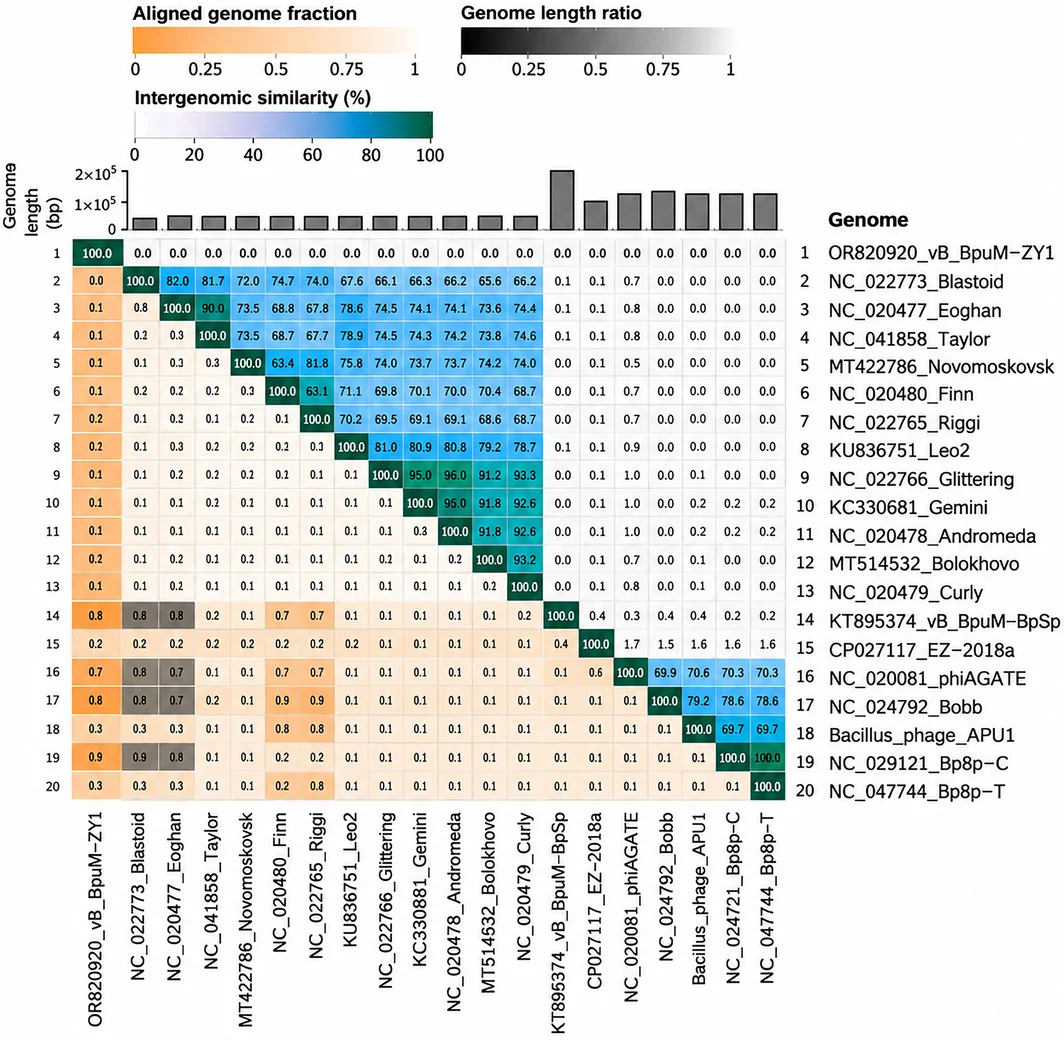
VIRIDIC heatmap showing intergenomic similarity among phage APU1 and related *Bacillus* phages. APU1 shares highest identity with *Bp8p‐C* (89.74%) and *Bp8p‐T* (89.75%), below the 95% ICTV species threshold (dashed line). Lower identities (< 80%) appear in yellow–white.

Intergenomic similarity assessed with VIRIDIC (Figure 4) demonstrated that phage APU1 shares 89.75% and 89.74% average nucleotide identity (ANI) with *Bacillus* phages Bp8p‐T and Bp8p‐C, respectively—values below the ICTV's 95% species delineation threshold. This confirms that APU1 represents a genomically distinct variant within the same genus.

**FIGURE 5:**
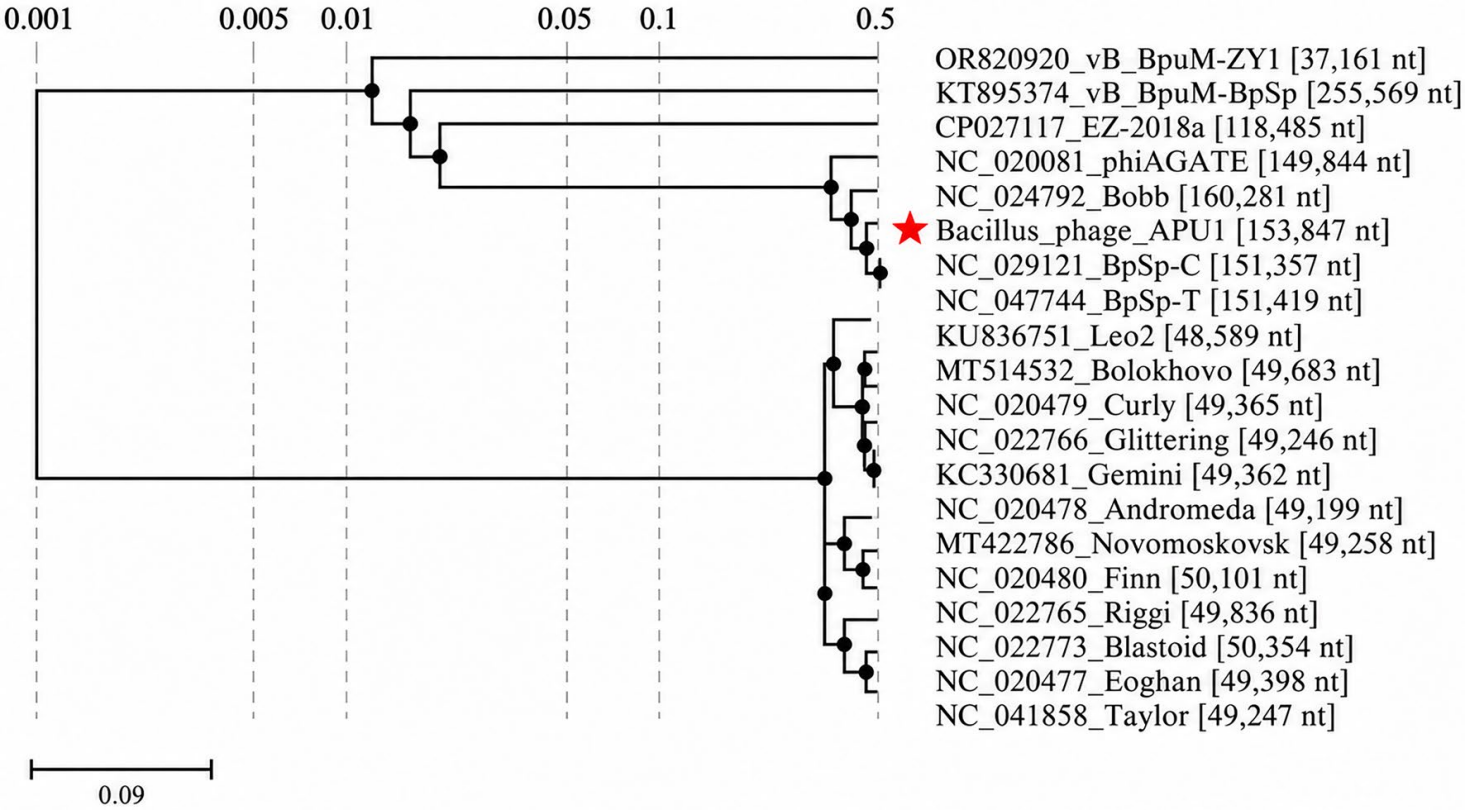
VIPTree proteomic phylogeny of phage APU1 and related *Bacillus* phages. The tBLASTx‐based tree shows APU1 clustering with phages Bp8p‐C (NC_029121), Bp8p‐T (NC_047744) and Bobb (NC_029128), forming a cohesive genus‐level group. Branch lengths reflect evolutionary distance, with scale bar indicating 0.2 substitutions per site. Bootstrap values > 90% support all major nodes.

**FIGURE 6:**
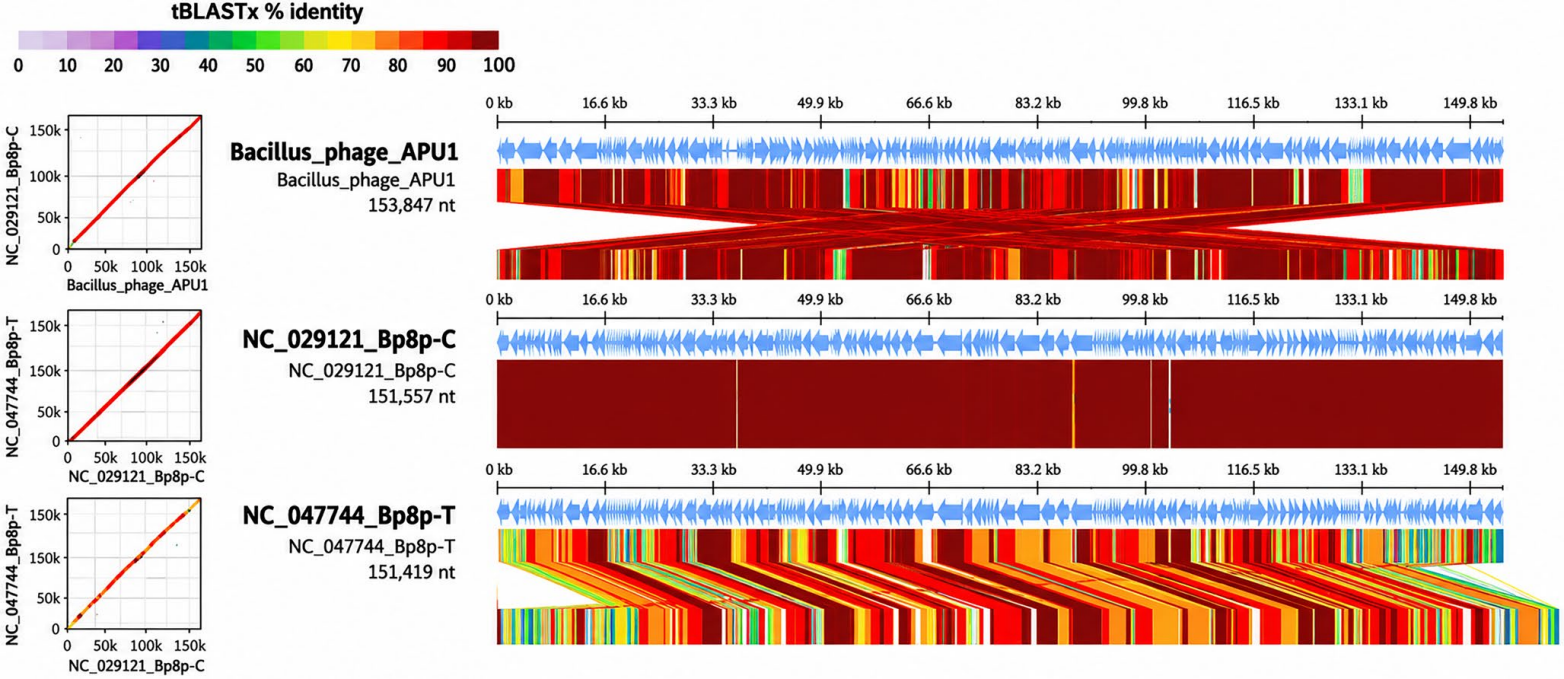
Whole‐genome alignment of phage APU1 with related *Bacillus* phages Bp8p‐C and Bp8p‐T. Pairwise tBLASTx comparisons show sequence identity from 40% (tan) to 100% (dark red). Arrows indicate predicted coding sequences and their orientation. The genomes display high overall synteny with localised rearrangements and regions of reduced identity (< 85%).

Proteome‐based phylogeny inferred from VIPTree (Figure 5) revealed strong proteomic similarity between phage APU1 and its closest relatives (Bp8p‐C, Bp8p‐T and Bobb), with pairwise proteomic similarity scores (*S*_h_ > 0.15) exceeding the genus‐level threshold established by ICTV guidelines. The proteomic relationships were further validated by whole‐genome alignment (Figure 6), which demonstrated conserved synteny across all four phages, with minor genomic rearrangements in APU1 and interspersed regions of high nucleotide identity (> 85%) with divergent segments. Collectively, these results—integrating VICTOR (whole‐proteome distances), VIRIDIC (genome‐level ANI), and VIPTree (proteomic similarity)—provide consistent evidence that APU1 is a genomically distinct member of an established *Bacillus* spp.‐infecting phage genus, contributing to the expanding diversity recognised within this lineage.

## 4 Discussion

The characterisation of bacteriophages from underexplored environments is crucial for advancing our understanding of viral diversity and for building repositories that may support future biocontrol studies. This study presents the high‐quality assembled genome and comparative analysis of APU1, a bacteriophage isolated from Bangladeshi soil that infects *Bacillus* sp. The genome showed a distinct genomic signature within a group of related *Bacillus* phages and provides additional insight into the diversity of large Caudoviricetes phages from soil environments. APU1 showed genomic features consistent with a predicted lytic lifestyle. PhaBOX classified APU1 as virulent, with PhaTYP and CHERRY prediction scores of 1.0, and manual inspection of the final annotation did not identify canonical integrase, excisionase, CI repressor or Cro repressor genes. Therefore, this finding is interpreted as computational evidence supporting a predicted lytic lifestyle. In addition, CARD/VFDB screening through the PhageScope workflow did not detect CARD‐listed antimicrobial resistance genes or VFDB‐listed virulence factor genes under the applied thresholds.

The genome's modular organisation into functional clusters for replication, structural assembly and host lysis mirrors the efficient, specialised design seen in other virulent *Bacillus* phages like phiAGATE (Barylski et al. 2014) and those within the Spounavirinae subfamily. Comparative genomic analysis places APU1 within a recognised genus of *Bacillus* sp.‐infecting phages. While VICTOR and VIPTree analyses show clear proteomic clustering with phages Bp8p‐C, Bp8p‐T and Bobb, the intergenomic similarities calculated by VIRIDIC are definitive. The ANI values of 89.7%–89.8% with its closest relatives are below the 95% threshold used for species demarcation (Moraru et al. 2020), confirming APU1 as a distinct species within this genus. This genomic divergence suggests evolutionary adaptation, potentially involving the unique complement of five tRNAs and the set of 51 long hypothetical proteins identified, whose functions in host interaction or replication efficiency merit further investigation.

The primary significance of this work lies in its contribution to a sparse genomic landscape. As noted, *Bacillus*‐infecting phages are poorly represented in databases (Chen et al. 2023; Fard et al. 2010). Isolating and characterising phages from diverse geographical and ecological niches, such as the agricultural soil sampled here, is fundamental to mapping the true diversity of the viral world and understanding phage‐host co‐evolution. Phages from local environments may possess adaptations relevant to managing bacterial populations in similar settings. While not a clinical isolate, the resilience of *Bacillus* sp. in industrial and environmental contexts makes phages like APU1 relevant for exploring non‐antibiotic‐based control strategies in the future (Fu et al. 2020; Skorynina et al. 2022).

We acknowledge the limitations of this in silico characterisation. The enrichment host was conservatively identified as *Bacillus* sp. based on partial 16S rRNA gene sequencing, because multiple *Bacillus* species produced equivalent top BLASTn matches. The lytic activity and host range were conducted against a single host strain from our laboratory collection. A broader host range assay against a panel of diverse *Bacillus* sp. strains, including those from environmental and clinical settings, is necessary to assess its potential application scope. Virion morphology was not assessed by TEM or SEM because electron microscopy facilities were not available; therefore, capsid morphology, tail structure and virion dimensions remain to be experimentally confirmed. In addition, genome termini were not experimentally validated, and one‐step growth curve, adsorption kinetics and pH/temperature stability assays were not performed. These experiments should be prioritised in future work to better define the biological properties and applied potential of APU1. Furthermore, the functional predictions for the numerous hypothetical proteins and the biological implications of the unique tRNA set remain untested. In conclusion, APU1 is a *Bacillus* phage isolated from Bangladeshi soil with a high‐quality assembled genome and a computationally predicted lytic lifestyle. Its value lies in its detailed genomic characterisation, which clarifies its taxonomic position and provides a foundation for future research. Subsequent work should focus on experimental validation of its host range, structural analysis of its gene products and functional studies to decipher the roles of its unique genetic features.

## Author Contributions

**Arefeen Haider:** conceptualization, methodology, software, data curation, formal analysis, investigation, validation, visualization, writing – original draft. **Tasnuva Jahan Esha:** investigation, data curation, formal analysis, validation. **Sezanur Rahman:** writing – review and editing, methodology. **Jannat Akther:** methodology, writing – review and editing. **Naimul Islam Faysal:** formal analysis, validation. **Tahsin Khan:** formal analysis, validation. **Shakhinur Islam Mondal:** writing – review and editing. **Anowara Begum:** writing – review and editing. **Sudhangshu Kumar Biswas:** writing – review and editing. **Mustafizur Rahman:** conceptualization, methodology, supervision, resources, funding acquisition, writing – review and editing, project administration. **Mohammad Jubair:** conceptualization, writing – original draft, writing – review and editing, resources, supervision, funding acquisition, project administration, methodology.

## Funding

The authors have nothing to report.

## Disclosure

The corresponding author affirms that this manuscript is an honest, accurate and transparent account of the study being reported; that no important aspects of the study have been omitted; and that any discrepancies from the study as planned (and, if relevant, registered) have been explained.

## Ethics Statement

This environmental study involved soil sample collection and analysis, with no human or animal subjects. Ethical approval was not required. Sampling followed local regulations, and permissions were obtained from landowners and authorities.

## Conflicts of Interest

The authors declare no conflicts of interest.

## Supporting information


**Table S1:** Genomic annotation and predicted functions of coding sequences (CDSs) identified in *Bacillus* phage APU.

**Table S2:** InterProScan‐based functional and domain annotation of predicted proteins encoded by *Bacillus* phage APU.

## Data Availability

The raw sequencing reads for *Bacillus* phage APU1 have been submitted to the NCBI Sequence Read Archive under BioProject accession PRJNA972335, BioSample accession SAMN48576476 and SRA accession SRR33749510. The assembled genome sequence of *Bacillus* phage APU1 has been assigned GenBank accession number PZ809441. The partial 16S rRNA gene sequence of the bacterial enrichment host used for APU1 isolation has been assigned GenBank accession number PZ794753.
